# Effect of high-quality nursing on neurological function psychological moods quality of life of elderly patients with stroke

**DOI:** 10.3389/fneur.2023.1259737

**Published:** 2023-10-16

**Authors:** Na Gao, Yaqiang Li, Changru Sang, Jiale He, Congxia Chen

**Affiliations:** ^1^Department of Neurology, First Affiliated Hospital of Anhui University of Science and Technology (First People's Hospital of Huainan), Huainan, China; ^2^Department of Neurology, People's Hospital of Lixin County, Bozhou, China

**Keywords:** acute cerebral infarction, negative psychological mood, quality of life, high-quality nursing, depression

## Abstract

**Objectives:**

The primary objective of the present investigation was to meticulously examine the efficacy of high-quality nursing care (HQN) on neurological restoration, amelioration of adverse psychological states, and augmentation of quality of life in geriatric patients diagnosed with acute cerebral infarction (ACI).

**Methods:**

A cohort of 240 patients, afflicted by ACI and admitted to our healthcare institution between February 2020 and March 2023, were incorporated into this longitudinal prospective analysis. Employing a random number table methodology, the patient cohort was bifurcated into a control group (*n* = 120) receiving conventional care and an observation group (*n* = 120) receiving HQN. Comparisons were conducted between the two cohorts concerning neurological functionality [as quantified by the National Institutes of Health Stroke Scales (NIHSS) and Barthel Index (BI) scores], psychological wellbeing [utilizing the Self-Rating Anxiety Scale (SAS) and Self-Rating Depression Scale (SDS) scores], overall quality of life [assessed via the Generic Quality of Life Inventory-74 (GQOLI-74) scores], and self-perceived burden [evaluated through the Self-Perceived Burden Scale (SPBS)]. Further assessments included patient satisfaction and incidence of complications, both in the pre- and post-interventional phases.

**Results:**

Post-intervention, the observation group demonstrated superior outcomes compared to the control group, as evidenced by diminished NIHSS and SPBS scores and elevated BI metrics. Moreover, SAS and SDS scores in both groups manifested a decline post-intervention; however, the decrement was statistically more pronounced in the observation group (*P* < 0.05). Similarly, all dimensions of GQOLI-74 showed an upward trend in both cohorts, yet the increase was significantly more substantial in the observation group (*P* < 0.05). Furthermore, the observation group exhibited a reduced frequency of complications coupled with heightened levels of nursing satisfaction.

**Conclusion:**

The implementation of HQN in the geriatric population afflicted by ACI markedly enhances neurological recuperation, attenuates adverse psychological states, and ameliorates overall quality of life. The intervention is also associated with a diminution in complication rates and an increase in nursing satisfaction, thereby substantiating its clinical utility.

## 1. Introduction

Cerebrovascular accidents, colloquially known as strokes, stand as a formidable cause of morbidity and mortality, contributing to nearly 5% of all debilitating diseases and accounting for 10% of global fatalities (1). Given its ubiquity, stroke retains its rank as the second most prevalent instigator of both disability and mortality on a global scale, with a disproportionate impact on low- and middle-income countries (2). Recent epidemiological data divulge that 13.7 million new stroke events were recorded globally in 2016, of which a staggering 87% were ischemic in nature, exhibiting a predilection for recurrence (3). Acute cerebral infarction (ACI), a subtype of ischemic stroke, is engendered by the necrotic or softening transformation of cerebral tissue as a consequence of ischemia and hypoxia. Clinically, its management is orchestrated via interventions focusing on cerebral circulation optimization, neuroprotection, modulation of intracranial pressure, and intravenous thrombolysis (4, 5). Despite these therapeutic approaches, a high incidence of recrudescence and adverse prognostic outcomes persist, imperiling patient survival. These infarcts predominantly result from an obstruction in cerebral blood flow, culminating in ischemic necrosis and subsequent cerebral infarction (6). The irrevocable nature of neuronal loss contributes to the elevated rates of disability and relapse associated with ACI. Compounding this issue are the demographic shifts and lifestyle alterations that have escalated the incidence of ACI among the elderly, thereby deteriorating their quality of life and inflicting significant emotional, financial, and psychological burdens on their families (7, 8). Furthermore, patients suffering from acute ischemic stroke (AIS) frequently display deficient disease management capabilities, attenuated treatment compliance, and limited access to efficacious post-discharge nursing interventions, thereby accentuating the risk of subsequent stroke events. Accordingly, the discovery and implementation of effective and secure nursing interventions within the clinical milieu remain paramount.

High-quality nursing (HQN) is conceptualized as an exemplary paradigm of patient-centered healthcare, aimed at delivering the zenith of qualitative nursing experiences via meticulous physical care and psychological guidance. This model not only epitomizes the humanistic ethos of nursing but also augments the scope and comprehensiveness of nursing services (9). Moreover, HQN is intrinsically aligned with the philosophy of holistic healthcare, bolstering the main axis of clinical nursing services and rendering it highly specialized as a methodical and comprehensive model of care. This particular framework of nursing has found widespread application across various specialized departments, including but not limited to neurology and otolaryngology, and has yielded favorable outcomes. Studies conducted by Gui et al. (10) elucidated that HQN interventions manifest significant amelioration in elderly patients with Parkinson's disease by mitigating negative affective states, enhancing quality of life, and accelerating motor function recovery. Additionally, HQN measures have proven efficacious in patients subjected to radiotherapy for nasopharyngeal carcinoma by augmenting treatment effectiveness, attenuating the incidence of adverse reactions, and amplifying patient awareness concerning healthcare knowledge. Such interventions concomitantly alleviate negative emotional states and enrich life quality, sleep patterns, and overall nursing satisfaction (11). Furthermore, research by Gong et al. (12) asserted that HQN significantly mitigates emotional distress and elevates the quality of life in patients afflicted with malignant gliomas, thereby enhancing overall patient satisfaction with the nursing care received.

Nevertheless, there exists a paucity of empirical research concerning the applicability of HQN within the context of elderly patients diagnosed with ACI. As such, the present study endeavors to implement HQN interventions for this demographic to scrutinize their impact on rehabilitation outcomes, psychological wellbeing, and quality of life, thereby establishing the feasibility of this model as a prospective nursing intervention program.

## 2. Materials and methods

### 2.1. Subjects

#### 2.1.1. Data collection and assessment

All subjects enlisted in this prospective observational study were sequentially diagnosed with AIS at the First Affiliated Hospital of Anhui University of Science and Technology (also known as the First People's Hospital of Huainan) during the timeframe spanning from February 2020 to March 2023. Exclusion criteria encompassed individuals who (1) manifested severe disorders of consciousness; (2) were afflicted with concomitant severe pathologies such as acute infections, hepatic and renal impairments, autoimmune disorders, hematological or rheumatic maladies, and oncological malignancies; (3) exhibited pronounced dysarthria or aphasia; and (4) were non-compliant with follow-up assessments or missed scheduled visits. Ethical approval for this research was granted by the Institutional Ethics Committee of the First Affiliated Hospital of Anhui University of Science and Technology, and informed consent was procured from all participants or their legally authorized representatives, in accordance with the Declaration of Helsinki of 1975.

The inclusion criteria stipulated that participants should be (1) of an age > 60 years but <80 years and (2) clinically diagnosed with AIS, corroborated by imaging modalities. Ultimately, a cohort of 240 patients was constituted and randomized into an observation arm (*n* = 120) and a control arm (*n* = 120).

### 2.2. Conventional nursing

The control cohort received standard care protocols, which included (1) immediate hospital admission facilitated by the deployment of monitoring instrumentation for real-time tracking of vital physiological metrics, followed by expeditious identification and reporting of anomalies; (2) provision of either a liquid diet or enteral nutrition via gastric tube infusion, modulated by the gravity of the patient's clinical state and the manifestation of potential sequelae; (3) administration of foundational pharmacological agents for neural nourishment and intracranial pressure modulation, as delineated by medical prescriptions, accompanied by comprehensive education on medication nomenclature and administration protocols; (4) patient education regarding requisite diagnostic tests tailored to their pathology, augmented by routine hematological assessments to holistically evaluate their physiological condition; and (5) patients being guided in executing moderate physical mobilization to expedite metabolic processes and circulatory function once clinical stability was attained.

### 2.3. High-quality nursing

The HQN initiative was operationalized under the nomenclature of HQN, juxtaposed against the backdrop of conventional care standards. This framework encapsulates four cardinal dimensions:

(1) Psychological care: Among the elderly population suffering from cerebral infarction, there is a heightened susceptibility to adverse emotional states such as fear and depression. Effective communication channels are opened, through which empathy, understanding, and encouragement are conveyed. This humanistic approach aims to foster psychological equilibrium in patients. Concurrently, interdisciplinary cooperation with physicians is leveraged to deliver judicious therapeutic interventions. Patients are educated regarding treatment regimens and anticipated outcomes, thereby galvanizing their confidence and nurturing a propitious psychological milieu.
(2) Nutritional guidance: Medical personnel elucidate the salience of dietary regulation to patients and their immediate support systems. Instructions are proffered for the consumption of facilely digestible and nutritive sustenance, including vegetables, fruits, and whole grains while maintaining an equilibrated nutritional calculus across daily meals. In parallel, recommendations for abstinence from tobacco and alcohol are issued. Patients are also advised against ingesting irritant substances and encouraged to augment fluid intake for gastrointestinal preservation and constipation prophylaxis.
(3) Rehabilitation protocols: Rehabilitation therapists and the lead nurse apprise patients of ergonomically favorable postures, specifically designed to mitigate undue stress on affected limbs. A regimen of strategic positional changes is instituted, leveraging functional pads to maintain the affected limb in a biomechanically advantageous alignment. Exercise paradigms are tailored to the unique clinical presentation and needs of each patient, progressing from passive maneuvers to active exercises in a phased manner. These are intended to ameliorate local circulation and obviate muscle spasms or atrophy. Complementary to these protocols, either familial caregivers or professional attendants are enlisted to assure safety during the rehabilitative process. Speech therapists also contribute by facilitating specialized exercises aimed at restoring speech functionality, incorporating exercises focused on lip and tongue coordination, respiratory control, and articulatory precision.
(4) In the domain of online health education, the medical department possesses the capability to instantiate WeChat groups that are meticulously tailored to the patients' specific medical conditions and symptomatic presentations. These digital conglomerates are engineered to facilitate an interactive conduit between patients, their familial caregivers, and nursing personnel. Furthermore, this virtual milieu serves as an invaluable platform for patient-peer encouragement and support. The WeChat interface is utilized to disseminate critical medical information across several categories, encompassing “Pathological Knowledge Dissemination,” “Rehabilitative Strategies,” and “Domestic Care Protocols,” among others. During their inpatient sojourn within the hospital, nursing staff proactively advocate for participation in these WeChat groups, urging both patients and family members to engage with the digital content comprehensively. This strategy aims to continually augment their grasp and expertise concerning disease pathologies and rehabilitative care.

### 2.4. Observation indicators

Patients were instructed to return to the medical facility for a follow-up consultation at a juncture 3 months post-intervention. Prior to and subsequent to the intervention (upon admission and at the 3 month mark, respectively), pertinent assessment instruments were deployed; for those unable to comply with the designated follow-up timeline, the assessments were alternatively conducted by the nursing staff via telephonic consultations.

① Psychological parameters: The Self-rating Anxiety Scale (SAS) and Self-rating Depression Scale (SDS) were employed to quantify levels of anxiety and depression in the patient cohort (13). Each scale is comprised of 20 items, evaluated on a four-tiered scoring system ranging from 0 to 100; an escalating score is indicative of deteriorating psychological health.

② Quality of life metrics: The Generic Quality of Life Inventory-74 (GQOLI-74) was administered pre- and post-intervention, facilitating a comparative analysis between the control and experimental cohorts (14). This multidimensional scale elucidates four core facets: material wellbeing, physiological functionality, psychological states, and social integration. The scoring metrics vary for each dimension, ranging from 16 to 80 for the physical life facet and 20 to 100 for the remaining three. The score is directly proportional to the individual's perceived quality of life.

③ Neurological functionality: Utilizing the National Institutes of Health Stroke Scale (NIHSS), neurological performance was gauged in both cohorts before and after the intervention (15). A higher NIHSS score correlates with compromised neurological integrity.

④ Activities of daily living (ADL): The Barthel Index (BI) was invoked to evaluate the patients' competencies in executing routine activities (16). The comprehensive score spans from 0 to 100, with a higher score emblematic of superior functional autonomy in daily living.

⑤ Self-perceived burden: The Self-Perceived Burden Scale (SPBS) was applied post-intervention to gauge the patients' subjective perception of burden across physical, emotional, and financial dimensions (17). The instrument incorporates 10 scored items, cumulatively amounting to a maximum of 50 points; a higher score underscores elevated self-perceived burden.

⑥ Patient satisfaction: A bespoke “Patient Clinical Care Satisfaction Questionnaire” was utilized to appraise the level of patient satisfaction post-intervention in both cohorts. This evaluative tool incorporates five items, with an aggregate score reaching up to 100. An elevated score indicates heightened levels of satisfaction concerning the nursing care received. Additionally, incidences of clinical complications subsequent to the intervention were meticulously documented and subjected to inter-group comparative analysis.

### 2.5. Statistical analysis

Statistical analyses were conducted employing SPSS for Windows (version 26.0, Inc., Chicago, IL, USA). For continuous variables conforming to a normal distribution, descriptive statistics were articulated in terms of means accompanied by standard deviations. In instances where the distribution was non-normal, medians along with interquartile ranges (IQR) were reported. Categorical variables were delineated by the utilization of percentages and absolute frequencies. The Kolmogorov-Smirnov test served to evaluate the normality of the distributions under investigation. Comparative analyses for continuous variables with a normal distribution were executed via the Student's *t*-test. Conversely, continuous variables that deviated from normality were subjected to the Mann-Whitney *U*-test. Categorical variables underwent comparative evaluation between groups through the application of Fisher's exact test or Pearson's chi-square (χ^2^) test, as pertinent to the data type. For the evaluation of differences between groups concerning continuous variables with non-normal distributions, the Mann-Whitney *U*-test served as the chosen statistical instrument. A *P*-value of <0.05 was established as the threshold for statistical significance.

## 3. Results

### 3.1. Clinical and demographic characteristics of patients in the observation and control groups

The study enrolled a cohort of 240 patients, comprising 120 individuals in the control group (of whom 71 were women) and 120 individuals in the observation group (of whom 68 were women). The mean age of the control group was 69.97 ± 7.66 years, while the mean age of the observation group was 69.57 ± 5.15 years. Statistical analysis revealed no significant age-related difference between the two groups (*P* > 0.05). Similarly, other baseline attributes did not exhibit statistically significant variances between the control and observation groups, as depicted in Table 1.

**Table 1:** Clinical and demographic characteristics of patients in the observation and control groups.

**Variables**	**Observation group (*N* = 120)**	**Control group (*N* = 120)**	***t-*value, Z-value or *χ^2^* value**	***P*-value**
**Demographic characteristics**				
Sex, female, *n* (%)	71 (59.17)	68 (56.67)	0.154	0.695
Age, years, mean ± SD	69.57 ± 5.15	69.97 ± 7.66	−0.472	0.637
Education years, median (IQR)	5 (3–7)	5 (3–8)	−1.131	0.157
Married, *n* (%)	115 (95.83)	118 (98.33)	1.324	0.250
**Vascular risk factors (%)**				
Hypertension	96 (75.21)	90 (76.06)	0.860	0.354
Diabetes mellitus	52 (43.33)	56 (46.67)	0.269	0.604
Coronary heart disease	19 (15.83)	15 (12.5)	0.548	0.459
Atrial fibrillation	12 (10)	15 (12.5)	0.376	0.540
current smoking	42 (35)	40 (33.33)	0.074	0.785
Alcohol consumption	32 (26.67)	29 (21.67)	0.198	0.656
TOAST subtype (*n* %)			0.391	0.822
Large artery atherosclerosis	59 (59.17)	53 (44.17)		-
Cardioembolism	12 (10)	15 (12.5)		-
Small vessel occlusion	49 (40.83)	52 (43.33)		-

### 3.2. The SAS and SDS scores between the two groups of patients

**Figure 1:**
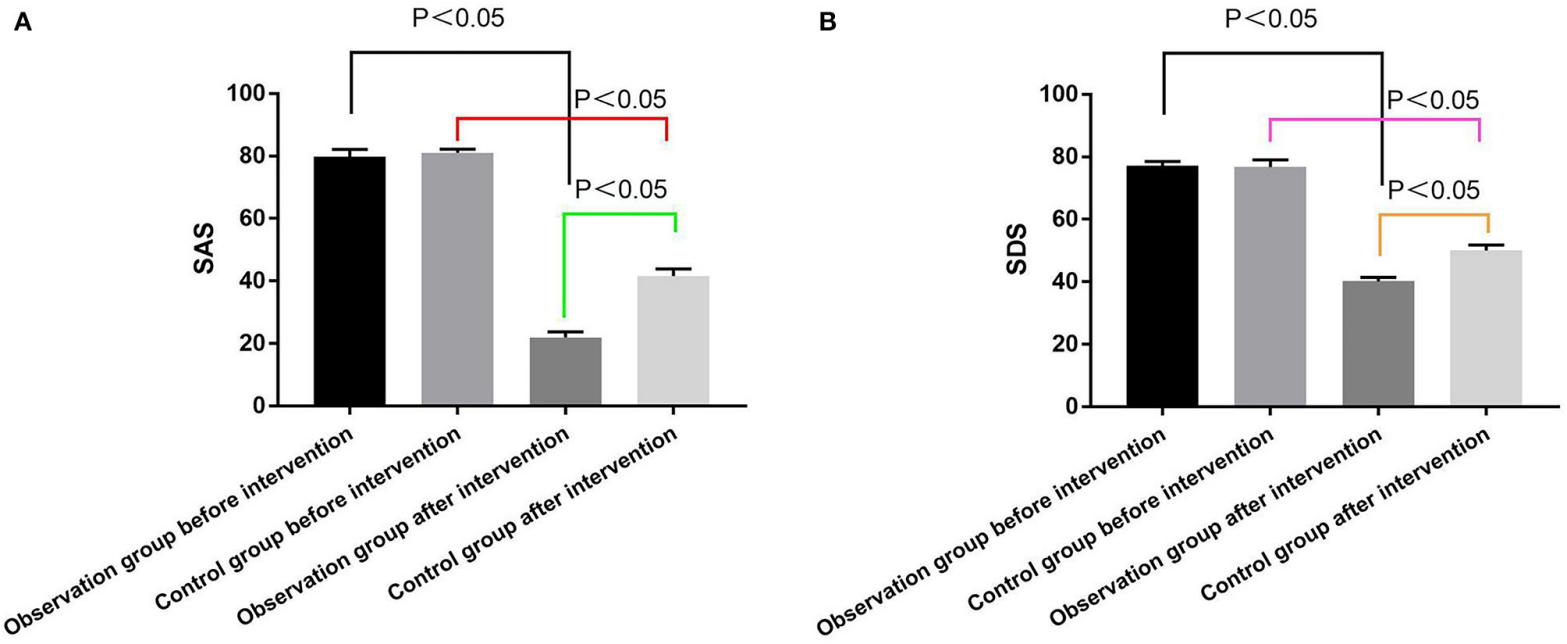
Comparison of the SAS and SDS scores. **(A)** SAS scores of the two groups before and after intervention; **(B)** SDS scores of the two groups before and after intervention.

Negative emotional states in both groups were assessed using the SAS and SDS during pre- and post-intervention. Prior to the intervention, no substantial differentiation was observed in the SAS and SDS scores between the two groups (*P* > 0.05). Subsequent to the intervention, noteworthy improvements in SAS and SDS scores were registered in both cohorts. However, these enhancements were particularly pronounced in the observation group (*P* < 0.05) (Figure 1).

### 3.3. Comparison of GQOLI-74 scores between the two groups

In the observation cohort, the patients' pre-intervention evaluations across the dimensions of social function, physical function, psychological function, and material life, as measured by the GQOLI-74, were 61.33 ± 2.87, 62.82 ± 2.95, 64.33 ± 2.64, and 61.25 ± 3.25, respectively. Correspondingly, the post-intervention assessments yielded scores of 88.64 ± 3.23, 87.86 ± 3.82, 89.45 ± 3.12, and 87.61 ± 2.36. Conversely, in the control cohort, the pre-intervention scores for these aforementioned dimensions were 61.12 ± 2.93, 62.53 ± 2.73, 64.66 ± 2.52, and 61.03 ± 3.08; the post-intervention metrics stood at 72.86 ± 2.71, 70.32 ± 2.54, 71.46 ± 2.67, and 73.21 ± 3.22.

Prior to the intervention, the statistical evaluation revealed no significant disparities in any of the four dimensions—namely social functioning, physical functioning, psychological functioning, and material life—between the observation and control cohorts (*P* ≥ 0.05). Subsequent to the intervention, enhancements in all GQOLI-74 dimensions were evident in both groups; however, the observation cohort manifested statistically superior improvements in all dimensions under consideration (*P* < 0.05) (Table 2).

**Table 2:** Comparison of GQOLI-74 scores before and after intervention.

**Time**	**Group**	**Social function**	**Physical function**	**Psychological function**	**Material life**
Before intervention	Observation group (*n* = 120)	61.33 ± 2.87	62.82 ± 2.95	64.33 ± 2.64	61.25 ± 3.25
	Control group (*n* = 120)	61.12 ± 2.93	62.53 ± 2.73	64.66 ± 2.52	61.03 ± 3.08
	*t*-value	0.510	0.639	0.362	0.347
	*P*-value	0.306	0.262	0.359	0.365
After intervention	Observation group (*n* = 120)	88.64 ± 3.23	87.86 ± 3.82	89.45 ± 3.12	87.61 ± 2.36
	Control group (*n* = 120)	72.86 ± 2.71	70.32 ± 2.54	71.46 ± 2.67	73.21 ± 3.22
	*t*-value	27.036	30.977	26.464	25.505
	*P*-value	0.000	0.000	0.000	0.000

### 3.4. Comparison of NIHSS and BI scores between the two groups

In the control cohort, the NIHSS scores of patients prior to and post-intervention were [9 (7–12)] and [6 (4–8)], respectively. Correspondingly, the BI scores were [40 (30–50)] and [50 (45–70)], respectively. Within the observation cohort, the NIHSS scores pre- and post-intervention were [9 (8–11)] and [5 (4–7)], respectively; the BI scores followed the same sequence, manifesting as [40 (30–50)] and [60 (50–80)]. The data represented in Table 3 underscored no statistically significant divergence in NIHSS and BI scores between the cohorts upon admission (*P* > 0.05). Subsequent to the intervention, the observation cohort demonstrated decreased NIHSS scores and elevated BI scores compared to the control cohort, attaining statistical significance (*P* < 0.05).

**Table 3:** Comparison of NIHSS scores and Barthel Index scores between the two groups.

**Group**	**NIHSS scores**		**Barthel Index scores**	
	**At the time of admission**	**After intervention**	**At the time of admission**	**After intervention**
Observation group	9 (8–11)	5 (4–7)	40 (30–50)	50 (40–80)
Control group	9 (7–12)	6 (4–8)	60 (55–70)	40 (30–70)
*P-*value	0.133	0.027	0.188	0.031
Z-value	1.212	−2.123	−9.331	5.431

### 3.5. Comparison of SPBS scores between the two groups after the intervention

In the control cohort, the metrics for emotional, physical, and economic dimensions on the SPBS (Scale of Psychological, Physical, and Economic Burden) were 17.18 ± 2.27, 24.25 ± 1.62, and 7.17 ± 1.08, respectively. In the observation cohort, these dimensions correspondingly registered 13.41 ± 2.92, 18.11 ± 2.74, and 3.99 ± 0.74. Post-intervention, the observation cohort presented statistically significant reductions across all three SPBS dimensions (*P* < 0.05), as delineated in Table 4.

**Table 4:** Comparison of SPBS scores between the two groups of patients after intervention.

**Group**	**Emotional dimension**	**Physical dimension**	**Economic dimension**
Observation group	13.41 ± 2.92	18.11 ± 2.74	3.99 ± 0.74
Control group	17.18 ± 2.27	24.25 ± 1.62	7.17 ± 1.08
*t*-value	−11.158	−21.101	−26.529
*P-*value	0.000	0.000	0.000

### 3.6. Comparison of complication rates between the two groups

The observation cohort experienced nine serious complications, namely two instances of gastrointestinal bleeding, one intracranial hemorrhage, two pulmonary infections, two mortalities, one urinary tract infection, and one deep vein thrombosis. Conversely, the control cohort reported four cases of gastrointestinal bleeding, three intracranial hemorrhages, four pulmonary infections, two mortalities, five urinary tract infections, and one deep vein thrombosis. These data, depicted in Table 5, substantiate that the observation cohort had a lower overall rate of complications, achieving statistical significance (*P* < 0.05).

**Table 5:** Comparison of complication rates between the two groups.

**Group**	**Observation group (*N* = 120)**	**Control group (*N* = 120)**	***χ^2^* value**	***P*-value**
Gastrointestinal bleeding	2 (1.67)	4 (3.33)	-	-
Intracranial hemorrhage	1 (0.83)	3 (2.5)	-	-
Pulmonary infection	2 (1.67)	4 (3.33)	-	-
Death	2 (1.67)	2 (1.67)	-	-
Urinary tract infections	1 (0.83)	5 (4.17)	-	-
Deep vein thrombosis	1 (0.83)	1 (0.83)	-	-
Total incidence	9 (7.5)	19 (15.83)	4.043	0.044

### 3.7. Comparison of nursing satisfaction between the two groups

In the observation cohort, 90 patients reported high levels of satisfaction with the nursing care, 25 were satisfied, and five were dissatisfied, culminating in a nursing satisfaction rate of 95.83%. In contrast, the control cohort included 80 highly satisfied patients, 15 satisfied, and 22 dissatisfied, amounting to a nursing satisfaction rate of 81.67%. The enhanced nursing satisfaction rate in the observation cohort compared to the control cohort was statistically significant (*P* < 0.05), as corroborated by Table 6.

**Table 6:** Comparison of the nursing satisfaction [*n* (%)].

**Group**	**Observation group (*n* = 120)**	**Control group (*n* = 120)**	***χ^2^* value**	***P*-value**
Very satisfied	90 (75)	80 (66.67)	-	-
Satisfied	25 (20.83)	18 (15)	-	-
Dissatisfied	5 (4.17)	22 (18.33)	-	-
Nursing satisfaction	115 (95.83)	98 (81.67)	12.061	0.001

## 4. Discussion

To the best of our scholarly understanding, the present investigation constitutes an inaugural exploration into the applicability of HQN for geriatric populations afflicted with ACI. ACI serves as a recurrent clinical emergency within elderly demographics, displaying elevated rates of morbidity and mortality and consequently exerting a profound impact on the overall quality of life for both patients and their familial units. Furthermore, due to the advancing age of the majority of ACI patients, a concomitant physiological decline renders conventional caregiving paradigms increasingly ineffectual. As such, the exigencies surrounding clinical nursing care for these patients have become a salient focus within the medical community. In parallel, the accelerated economic development of China has engendered an escalating demand for comprehensive healthcare services. Contemporary patients manifest expectations that extend beyond mere therapeutic efficacy; they increasingly prioritize comfort and patient-centricity throughout the continuum of medical treatment. In response, leading healthcare institutions are actively engaged in ongoing reformative initiatives targeted at optimizing both the quality and operational efficiency of nursing services.

Negative psychological affectivity, an umbrella term encompassing anxiety, depression, tension, and pain, arises when situational or individual expectations diverge from actual outcomes (18). Within the context of ACI, patients often experience heightened levels of anxiety and depression due to the formidable medical costs associated with treating the condition and the consequent psychosocial burden on both themselves and their families (19, 20). Existing literature corroborates that such negative psychological affectivity can deleteriously influence patients' recuperative trajectories and exacerbate their overall medical conditions. Specifically, for patients who have experienced ACI, these adverse emotional states may not only precipitate recurrence but also impair neurological functionality, thereby entrapping the patient in a detrimental feedback loop (21).

Our empirical findings reveal that the SAS and SDS scores within the observation cohort were statistically lower in comparison to the control group post-intervention, suggesting that HQN plays a contributory role in attenuating negative psychological affectivity among elderly ACI patients. This ameliorative effect may be attributed to HQN's patient-centered nursing approach, which employs strategies such as empathy and relational transposition to strengthen the therapeutic alliance. Concurrently, meticulous and compassionate communication encourages patients to divest themselves of emotional encumbrances. Moreover, the attending nurse, having discerned the innermost concerns of the patient, is positioned to offer timely psychological counseling tailored to individual needs when negative emotions are detected.

Supporting this, Wu et al. (22) have ascertained that HQN significantly ameliorates anxiety and depression in acute stroke patients undergoing magnetic resonance imaging (MRI) procedures, not only enhancing examination completion rates but also reducing examination durations and augmenting patient satisfaction. Similarly, HQN interventions have proven efficacious in attenuating the psychological stress associated with initial chemotherapy sessions for oncology patients, thereby improving both psychological wellbeing and overall treatment efficacy (23). In a study conducted by Mei et al. (24) the implementation of HQN post-lung cancer surgery not only markedly improved patients' psychological wellbeing but also increased patient compliance and satisfaction while reducing postoperative complications and recurrence rates, findings that align synergistically with the results of the current study.

The NIHSS scores for the observation group were markedly lower subsequent to the intervention in comparison to the control group, while conversely, the BI scores were notably elevated. These empirical data substantiate that HQN exerts a significant positive influence on the neurological outcomes of patients diagnosed with ACI. A plausible explanation for this salutary effect is HQN's meticulous emphasis on the optimization of rehabilitation protocols, buttressed by a commitment to professional excellence in functional training modalities. Nurses attuned to HQN principles adaptively customized care regimens commensurate with the patient's stage of neurological recovery, ranging from motor function to linguistic capabilities, thereby fostering robust improvements in neurological functionality.

Notably, Huang et al. (25) demonstrated that a HQN-oriented approach incorporating low-frequency pulsed electrical stimulation in tandem with early whole-body functional exercise ameliorated limb functionality in patients suffering from brachial plexus injuries. Similarly, Narigele et al. (26) ascertained that HQN, when deployed in conjunction with the Montessori technique for clavicle fractures, significantly enhances both the quality of life and patient satisfaction while expediting joint functional recovery.

Patients afflicted with ACI typically undergo protracted recovery timelines necessitating long-term pharmacological regimens, a scenario that often culminates in suboptimal treatment compliance. Remarkably, our study divulged that the observation cohort exhibited superior GQOLI-74 scores across all metrics relative to the control group (*P* < 0.05), implying that HQN not only ameliorates the quality of life in elderly patients with cerebral infarction but also fortifies their adherence to medication protocols. Corroborating this, extant literature has demonstrated that the synergistic application of HQN alongside health education within chemotherapy protocols for non-small cell lung cancer yields appreciable clinical benefits. Such an integrative approach not only enhances patient compliance and self-management but also exerts a salubrious impact on both their physical and psychological wellbeing (27).

The salient innovation of this study lies in its exhaustive analytical framework, encompassing multiple facets such as neurological functionality, psychological wellbeing, GQOLI-74 metrics, complication prevalence, and treatment satisfaction. Through this holistic lens, the study corroborates the efficacy and safety of HQN in the management of ACI, thus proffering an alternative therapeutic paradigm for this patient demographic. Future research endeavors ought to focus on elucidating the differential impacts of HQN across ischemic stroke subtypes, namely lacunar, atherothrombotic, cardioembolic, cryptogenic, and idiopathic infarctions. Such inquiries promise to furnish invaluable knowledge regarding the unique pathophysiological profiles, prognostic markers, and clinical symptomatology that distinguish lacunar strokes from other cerebral infarcts.

Despite its methodological rigor, the current investigation is not devoid of limitations and potential biases, particularly in the domain of sample selection. The cohort size for this study remains constrained, thereby necessitating future research that expands the sample size to substantiate the reliability of the findings in a more robust manner. Moreover, the duration of HQN intervention was delimited to a 3 month timeframe, resulting in a paucity of longitudinal data concerning ACI patients. Given these constraints, including the single-center scope and limited temporal breadth of the study, it remains imperative for subsequent iterations to both augment sample sizes and initiate multi-center, large-scale randomized controlled trials. This would serve to fortify the empirical foundation underlying the efficacy of HQN in geriatric patients diagnosed with ACI.

## 5. Conclusion

The HQN paradigm demonstrably exerts a salutary influence on multiple facets of patient wellbeing in the context of ACI. Notably, HQN contributes to the amelioration of neurological function, mitigates negative psychological states, fosters neurological recuperation, enhances overall quality of life and nursing satisfaction metrics, attenuates patient-perceived burden, and minimizes the incidence of complications. As such, the HQN model merits further application and dissemination within clinical settings.

## Data availability statement

The original contributions presented in the study are included in the article/supplementary material, further inquiries can be directed to the corresponding author.

## Ethics statement

The studies involving human participants were reviewed and approved by First Affiliated Hospital of Anhui University of Science and Technology (First People's Hospital of Huainan) (approval number: KJ2020A0023). Participants provide informed consent prior to inclusion in this study.

## Author contributions

YL: Conceptualization, Data curation, Formal analysis, Funding acquisition, Investigation, Methodology, Project administration, Resources, Software, Supervision, Validation, Visualization, Writing–original draft, Writing–review and editing. NG: Data curation, Investigation, Writing–original draft, Writing–review and editing. CS: Data curation, Formal analysis, Investigation, Writing–review and editing. JH: Data curation, Investigation, Writing–review and editing. CC: Data curation, Investigation, Writing–review and editing.
